# Different Culture Metabolites of the Red Sea Fungus *Fusarium equiseti* Optimize the Inhibition of Hepatitis C Virus NS3/4A Protease (HCV PR)

**DOI:** 10.3390/md14100190

**Published:** 2016-10-20

**Authors:** Usama W. Hawas, Radwan Al-Farawati, Lamia T. Abou El-Kassem, Adnan J. Turki

**Affiliations:** 1 Marine Chemistry Department, Faculty of Marine sciences, King Abdulaziz University, P.O. Box 80207, Jeddah 21589, Saudi Arabia; rfarawati@kau.edu.sa (R.A.-F.); aturki@kau.edu.sa (A.J.T.); 2 Pharmacognosy Department, National Research Centre, Dokki 12622, Cairo, Egypt; lamiaetaha@yahoo.com

**Keywords:** brown alga, *Padina pavonica*, Red Sea, *Fusarium equiseti*, HCV protease

## Abstract

The endophytic fungus *Fusarium equiseti* was isolated from the brown alga *Padina pavonica*, collected from the Red Sea. The fungus was identified by its morphology and 18S rDNA. Cultivation of this fungal strain in biomalt-peptone medium led to isolation of 12 known metabolites of diketopeprazines and anthraquinones. The organic extract and isolated compounds were screened for their inhibition of hepatitis C virus NS3/4A protease (HCV PR). As a result, the fungal metabolites showed inhibition of HCV protease (IC_50_ from 19 to 77 μM), and the fungus was subjected to culture on Czapek’s (Cz) media, with a yield of nine metabolites with potent HCV protease inhibition ranging from IC_50_ 10 to 37 μM. The Cz culture extract exhibited high-level inhibition of HCV protease (IC_50_ 27.6 μg/mL) compared to the biomalt culture extract (IC_50_ 56 μg/mL), and the most potent HCV PR isolated compound (Griseoxanthone C, IC_50_ 19.8 μM) from the bio-malt culture extract showed less of an inhibitory effect compared to isolated ω-hydroxyemodin (IC_50_ 10.7 μM) from the optimized Cz culture extract. Both HCV PR active inhibitors ω-hydroxyemodin and griseoxanthone C were considered as the lowest selective safe constituents against Trypsin inhibitory effect with IC_50_ 48.5 and 51.3 μM, respectively.

## 1. Introduction

Hepatitis C virus (HCV) infection is a global public health problem with an estimated approximately 200 million people worldwide at risk of developing life-threatening liver diseases, leading to liver fibrosis, liver cirrhosis, hepatocellular carcinoma, and ultimately death [1]. The HCV genome is a positive-strand RNA that contains a single large open reading frame encoding a polyprotein. During the replication of HCV, the protease NS3 possesses serine protease activity at its amino terminal and helicase function at its carboxyl terminal [2]. Due to its bifunctional role, NS3/4A protease (PR) becomes an attractive target for antiviral therapy [3,4]. Marine endophytes are microorganisms that spend the entire or part of their life cycle colonizing tissues of their host marine organism without causing apparent symptoms of disease. Their relationships with their hosts range from symbiotic to slightly pathogenic [5]. Moreover, marine endophytes are identified as a prolific source of biologically active small molecules and are considered to be a potential lead for the development of many new pharmaceutical agents [6,7]. 

A plethora of bioactive secondary metabolites produced by members of the genus *Fusarium*, can be harmful to humans or have potential in drug development [8]. Although *Fusarium equiseti* is considered a moderately aggressive fungus, it is capable of producing a vast range of bioactive secondary metabolites, which exhibit both phyto- and cytotoxicity. These metabolites include the trichothecenes, beauvericin, butenolide, equisetine, zearalenone, and fusarochromanone (FUSCHR) [9]. The later metabolite has been shown to have both stimulatory and inhibitory effects on various mammalian cells [10,11]. The effects of FUSCHR on the growth of human melanoma cells both in vitro and in vivo at low concentrations (0.1–1 nmol/L) were found to be cytotoxic to many melanoma cell lines [12]. Furthermore, the naturally occurring zearalenone (ZEA) produced by *F. equiseti* is a macrocyclic lactone with high binding affinity to oestrogen-responsive proliferation of MCF-7 cells and only low acute toxicity [13]. ZEA becomes particularly interesting because of its oestrogenic properties and its ability to accumulate at high levels in naturally infected cereal grain [14]. Additionally, its great diversity has been observed in metabolite amounts and composition among natural *F. equiseti* populations, where, FUSCHR and ZEA are the most commonly occurring [9]. 

Based on the screening model of HCV protease inhibitors, different culture extracts of the Red sea fungus *Fusarium equiseti* were found to be active and 21 secondary metabolites were isolated and identified from both culture extracts. The isolated compounds were identified based on the spectral analyses and comparison with the literature data. These compounds were also evaluated for their inhibitory effect on HCV NS3/4A protease using a SensoLyte™ 520 HCV protease assay kit, as well as their antimicrobial activity.

## 2. Results and Discussion

### 2.1. Characterization of Isolated Compounds

The identification of the isolated fungus *Fusarium equiseti* from Red Sea alga *Padina pavonica* was based on its morphology and authenticated by the molecular analysis of the internal transcript spacer (ITS1 and ITS4) region of rDNA, and the intervening 5.8S rDNA gene. The fungus was grown in a static biomalt-peptone liquid medium. The culture broth extract was evaluated for its inhibition of HCV NS3/4A protease and submitted for further chemical investigation of its secondary metabolites **1**–**12**. As a result of hepatitis C virus NS3/4A protease (HCV PR) bioassay, the fungal metabolites showed potent activity, and the fungus was subjected for further different culture optimization on Czapek’s (Cz) peptone media, with a yield of known metabolites **7**, **13**–**20**. A combination of silica gel column, preparative thin-layer, semi-preparative high performance liquid chromatography HPLC and Sephadex LH-20 column chromatography was used for isolation and purification of the active principle compounds.

**Figure 1:**
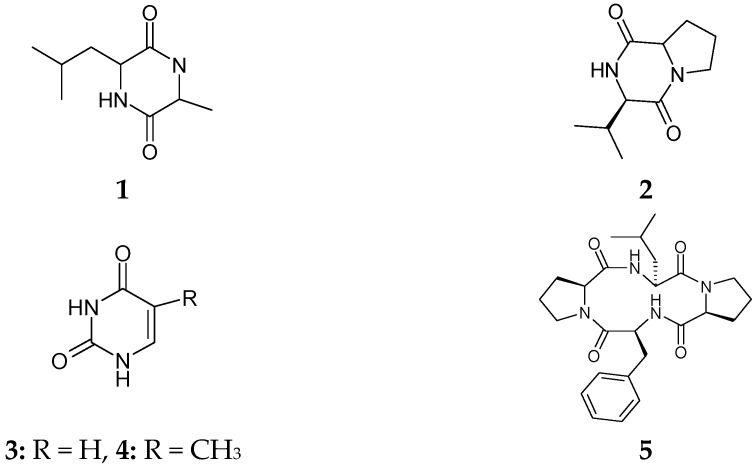

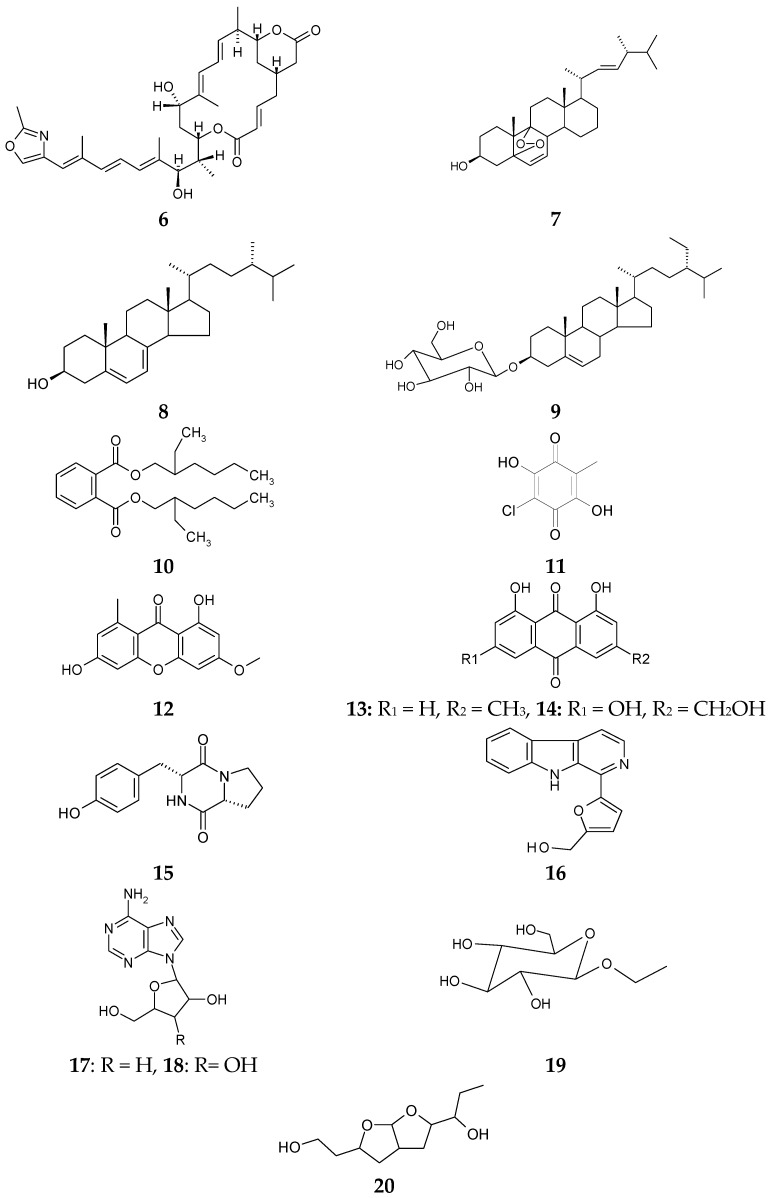
Structures of isolated compounds **1**–**20** from Red Sea *Fusarium equiseti* fungus.

Compounds **1**–**20** were detected by thin-layer chromatography (TLC) on silica gel as yellow, dark and blue spots under UV light. These UV absorbing spots were tentatively identified as anthraquinones, xanthones, adenosines, diketopiprazines, sugar and phenolic esters due to their colour reaction with KOH and Ehrlich’s reagents. The structures of all isolates (Figure 1) were elucidated on the basis of extensive NMR spectroscopy (1D- and 2D-NMR) and mass spectrometry (MS), as well as comparison with their literature data.

Alkaloid metabolites **1**–**6** and **15**–**18** with substituted nitrogen atoms showed both aliphatic (compounds **1** and **2**) and aromatic (compounds **3**–**6** and **15**–**18**) proton characters in their 1H- and ^13^C-NMR spectra. The compounds were characterized as diketopiprazines, cyclo-l-Ala-l-Leu (**1**) [15], cyclo(l-Pro-l-Val) (**2**) [16], cyclo(l-Tyr-l-Pro) (**15**) [17], uracil (**3**), thymine (**4**), cyclic tetrapeptidecyclo[Phenylalanyl-Pro-Leu-Pro] (**5**) [18]; perlolyrin (**16**) [19]; 17-demethyl-2,11-dideoxy-rhizoxin (**6**) [20] and two nucleosides, cordycepin (**17**) and ara-A (**18**) [21]. Bis-tetrahydrofurane derivative, communiol D (**20**) was reported as the fungal metabolite of *Podospora communis* [22]. There were *peri*-hydroxy quinones in compounds **11**–**14**, detected by their red colour with 5% KOH solution on TLC. The NMR spectra of compounds **12**–**14** were identified as tricyclic anthraquinones, griseoxanthone C [23], chrysophanol and ω-hydroxyemodin [24], respectively, and 5-Chloro-3,6-dihydroxy-2-methyl-1,4-benzoquinone (**11**) as a mono cylic quinone. Both culture extracts delivered steroidal compounds, ergosterol peroxide (**7**), ergostra-5,7-dien-3β-ol (**8**), β-sitosterol 3-*O*-β-glucoside (**9**) [25], in addition to bis(2-ethylhexyl)phthalate (**10**), and ethyl-*O*-β-glucoside (**19**). However, these compounds were isolated for the first time from Red Sea fungus *F. equiseti*.

### 2.2. Antimicrobial Activity

The antimicrobial activity of the EtOAc extracts and isolated compounds **1**–**20** of the fungus *F. equiseti* was evaluated against Gram positive bacteria and the fungus *Candida albicans* (Table 1). The results of the study indicated that the two extracts showed inhibitory activity against Gram-positive bacteria, *Bacillus subtilis, Staphylococcus aureus* and *Bacillus megaterium* as well as the fungus *Candida albicans*. The best antimicrobial action was found with *ω*-hydroxyemodin (**14**) and cordycepin (**17**) with inhibition zones ranging from 9 to 18 mm. 17-demethyl-2,11-dideoxy-rhizoxin (**6**) and ω-hydroxyemodin (**14**) showed the best activity towards the growth of *Candida albicans* with inhibition zone of 19 and 18 mm, respectively. *B. megaterium* was sensitive to all isolated tested compounds while *B. subtilis* was highly sensitive to cyclo(d-cis-Hyp-l-Leu) (**2**).

### 2.3. HCV NS3-4A Protease and Trypsin Inhibition Activities

HCV NS3 is considered as a serine protease, its active site contains serine 139 amino acid residues, which attack the substrate by forming a reversible covalent bond with its amino acid residues. To be activated, the HCV NS3 has to be coupled with an activating protein named (4A), and is therefore given the name HCV NS3/4A, which is active only in its heterodimer form. The culture extracts of the endophytic fungus *F. equiseti* and their isolated compounds were screened for inhibition of HCV protease using the hepatitis virus C NS3 protease inhibitor 2 as a positive control. Moreover, the selectivity of the active metabolites toward HCV NS3/4A protease (viral protease) and not human serine proteases such as trypsin and chymotrypsin has been confirmed through investigating the inhibitory activity of these extracts and/or their isolated chemical constituents on human recombinant Trypsin.

In Table 2, *F. equiseti* fungal metabolites from biomalt-peptone culture show good inhibition of HCV protease (IC_50_ from 19 to 77 μM). The isolated compounds griseoxanthone C (**12**) and cyclo(l-Pro-l-Val) (**2**) showed potent activity against HCV NS3/4A protease with IC_50_ values 19.8 and 23.2 μM, compared to their crude extract with IC_50_ value 56 μg/mL. Compounds cyclic tetrapeptidecyclo-[Phenylalanyl-pro-leu-pro] (**5**), 17-demethyl-2,11-dideoxy-rhizoxin (**6**), and 5-chloro-3,6-dihydroxy-2-methyl-1,4-benzoquinone (**11**) exhibited mild inhibitory effect with IC_50_ values of 29.4, 34.4, and 35.1 μM, respectively, while other compounds **1**, **4**, **8** and **9** were inactive as inhibitors of HCV PR as compared to other constituents. The fungus was subjected to different culture optimization on Cz media to yield nine known compounds, which exhibited high-level inhibition of HCV protease (IC_50_ from 10 to 37 μM), compared to the biomalt-peptone culture extract. The most potent HCV PR inhibitors were the isolates ω-hydroxyemodin (**14**) and cyclo(l-Tyr-l-Pro) (**15**) with IC_50_ values 10.7 and 18.2 μM, compared to their crude extract with IC_50_ value 27.6 μg/mL, respectively, while compounds cordycepin (**17**) and ara-A (**18**) exhibited a less potent inhibitory effect with IC_50_ values 22.3 and 24.5 μM, respectively. Perlolyrine (**16**) was considered as a mild inhibitor with IC_50_ value 37.8 μM.

The trypsins, which are serine proteases, underwent the most predominant genetic expansion yielding the enzymes responsible for digestion, blood coagulation, immunity, fibrinolysis, apoptosis, fertilization, and development [26,27]. This in vitro bioassay revealed that the Cz-culture extract exhibited mild inhibitory activity towards trypsin PR whereas biomalt-culture extract showed no inhibition effect at a concentration as high as 100 μg/mL. Accordingly, the selectivity of the ten compounds **2**, **5**, **6**, **11**, **12**, and **14**–**18** with relatively high activity against HCV protease was determined for their inhibitory activity on trypsin (Table 2). Only compounds **16** and **17** exerted any appreciable inhibitory activity against trypsin PR at a concentration up to 1 M, while compounds **2**, **12**, **14** and **15** showed moderate activity with an IC_50_ of 54.21, 51.33, 48.52 and 37.91 μM, respectively. These findings suggest that perlolyrine (**16**) and cordycepin (**17**), with moderate inhibitor activity on HCV PR, might not interfere with functional physiological reactions, and imply the relatively low toxicity of the compounds. Meanwhile, highly selective and potent HCV PR inhibitors griseoxanthone C (**12**) and ω-hydroxyemodin (**14**) are considered the lowest selective safe constituents.

### 2.4. Docking Study

The docking study of the HCV PR active compound **14** was used to formulate a hypothetical mechanism for the inhibitory activity of this active compound on the tested enzymes. This simulation established well the interactions between the HCV PR active site and conventional electrophile groups such as ketones and α-ketoamides, followed by trapping of the resulting covalently bound intermediate by the active site triad (His57, Ser139, and Asp81), which would provide effective inhibition. Moreover, the fitting of ligand functional groups to the shallow, solvent-exposed active site of the protease through other forces such as hydrogen-bonding and hydrophobic interactions plays an important role in the inhibition of the HCV PR [28]. In this study, the co-crystal structure ligand 2A4Q inhibits HCV PR by forming a reversible covalent bond between the carbonyl ketone of the inhibitor (code 2A4Q) and the hydroxy group of the enzyme active site Ser139, in addition to multiple hydrogen bonds with Gln41, Gly137, and Ala153 in the protease active site through its amide chain [29].

**Figure 2:**
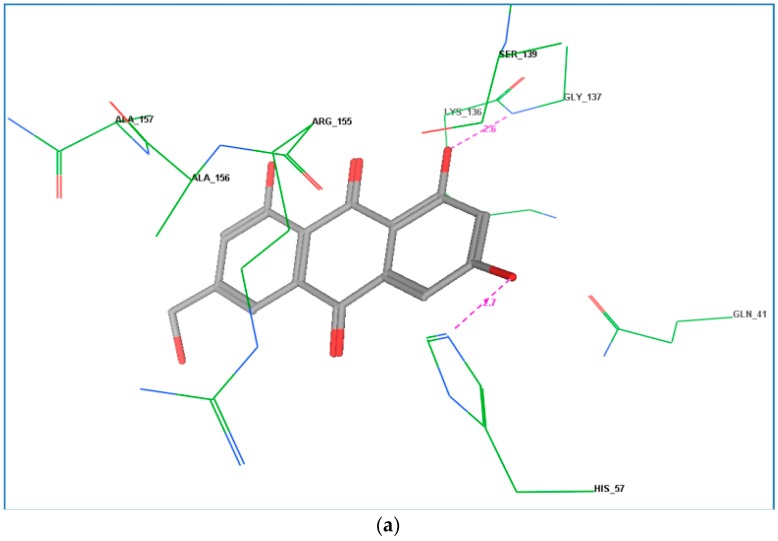

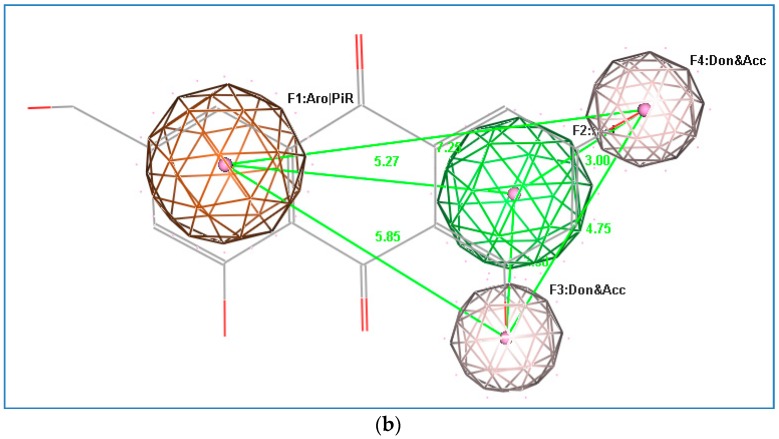
(**a**) Docking model of compound **14** to the HCV NS3-NS4A protease active site. H-bonds are represented by dashed lines, H-bonding length is measured in angstrom; (**b**) Superimposition of the pharmacophoric features on compound **14**.

The binding mode of ω-hydroxyemodin (**14**) showed interesting orientation via the formation of two hydrogen bonds between the *meta*-coupled phenolic hydroxyl groups and Gly137 and His57, one of the active triad amino acids in the active site, which could be one reason for its higher activity (Figure 2a). These docking results are in accordance with the proposed pharmacophore model regarding the significance of the hydroxy groups as H-bond donors and/or acceptors (Figure 2b). The active compound is predicted to form hydrogen bonds with amino acids other than Ser139, and the distance between the functional group (carbonyl ketone) and the hydroxy group of Ser139 was not sufficient to permit the formation of a covalent bond. This may be the reason why this compound is not potent as the positive control [29]. Thus, ω-hydroxyemodin (**14**) is introduced here as a promising lead HCV PR inhibitor for further semi-synthetic modification aiming at increasing its potency and selectivity.

## 3. Materials and Methods

### 3.1. General Experimental Procedures

Silica gel (60–120 mesh; Merck, Darmstadt, Germany) and Sephadex LH-20 (Pharmacia, Uppsala, Sweden) were used for Column Chromatography. Fluorescent TLC was used (Merck). *n*-Hexane, ethyl acetate and methanol were used for column chromatography. Mixtures of chloroform and methanol (5:95, 10:90 and 15:85, *v*/*v*) were used as mobile phase for TLC analysis, while chloroform and methanol (3:2 and 1:1, *v*/*v*) were used as mobile phase on Sephadex LH-20 column chromatography. Compounds were visualized as intense dark, blue and yellow coloured spots on TLC under UV. All compound spots changed after spraying with anisaldehyde/sulphuric acid followed by heating at 120 °C. NMR spectra were measured on a Bruker, Fallanden, Swtzerland) 600 (^1^H, 600 MHz; ^13^C, 150 MHz). Electron Spray Ionization mass spectrometry (ESI-MS) spectra were recorded on a Finnigan LCQ, San Jose, CA, USA) ion trap mass spectrometer.

### 3.2. Chemicals and Enzymes

Sensolyte™ 520 HCV Protease assay kit Fluorimetric (cat#AS-71145), HCV NS3/4A protease and Sensolyte™, Green protease Assay Kit Fluorimetric (cat#AS-71124), and Hepatitis Virus C NS3 protease inhibitor 2 (cat#AS-25346) were purchased from AnaSpec Inc., San Jose, CA, USA. Chymotrypsin inhibitor (Soybean trypsin) was purchased from Sigma Aldrich Co. (St. Louis, MO, USA), and Becton Dickinson Falcon™ (Tokyo, Japan). Microtest 384-well 120 μL black assay plates, no lid, non-sterile, were purchased from Becton Dickinson Inc, Franklin Lakes, NJ, USA. Culture media: Czapek agar, bacteriological peptone, malt extract, yeast extract powder, nutrient agar and potato dextrose broth were procured from Lab M, Bury, UK; Glucose, (Acros Organics, Geel, Belgium), K_2_HPO_4_ (Laboratory Reagent, Mumbai, India), MgSO_4_ (Oxford Laboratory Reagent, Mumbai, India), agar (Sisco Research Laboratories Pvt. Ltd., Mumbai, India).

### 3.3. Fungal Isolation and Culture Conditions

The brown alga sample of *Padina pavonica* was collected from the Egyptian Red Sea site at a depth of 3–5 m from the coast of South Hurghada in March 2010. In the laboratory, specimens were washed by sterile water and processed for identification by the Coral Reef Ecology and Biology group, National Institute of Oceanography and Fisheries, Suez, Egypt. Fungi *F. equiseti* was isolated as an epiphyte using potato dextrose agar (PDA) medium containing potato (200 g/L), glucose (10 g/L), and agar (15 g/L) at pH 7.5 prepared in 50% sea water supplemented with penicillin benzyl sodium salt (0.02 g/L) to avoid any bacterial growth. After 1–2 weeks white, velvety colonies were observed.

### 3.4. Identification of the Endophytic Isolates 

The pure isolated fungal strain *F. equiseti* was identified mainly by morphological methods by scrutinizing the culture, the mechanism of spore production, and the characteristics of the spores by macroscopic (colony morphology, texture, color, shape and size) and microscopic examination. The fungus was also subjected to DNA extraction and 18S rDNA sequence comparison.

After ten days of incubation in (PDA) medium, the mycelium of the fungus was scraped directly from the surface of the agar culture and weighed. Nucleic acid was extracted and purified using the GenElute™ DNA isolation kit for genomic DNA (Sigma Aldrich, St. Louis, MO, USA) using the Chomczynski method [30]. For identification of the fungal species, the internal transcript spacer regions (ITS1 and ITS4) and the intervening 5.8S rRNA region was amplified and sequenced using electrophoretic sequencing on 3130-genetic analyser (Fermentas, Glen Burnie, MD, USA; taq polymerase, dntps) using GenJET™ sequencing kit (Sigma Aldrich). The DNA fragment of the ITS regions was amplified using the polymerase chain reaction (PCR) with the pair of primers ITS1(5′-TTTTTTGGCGCATTCGTGTA-3′) and ITS4 (5′-CCCCATGCCCTTCAC-TGGGC-3′), and the intervening 5.8S rDNA region. Multiple sequence alignment and molecular phylogeny was performed using BioEdit [31]. According to sequencing similarities and multiple alignments, the fungus was found to be closely related to *Fusarium equiseti* (AF141949.1) with 99% identity. DNA sequencing was carried out by Sequencer Scientific Bourg El-Arab, Alexandria, Egypt.

### 3.5. Extraction and Isolation of Metabolites

The isolated fungus *F. equiseti* was cultivated on biomalt-peptone liquid medium (17 L) containing glucose (30 g/L), yeast (2 g/L), peptone (10 g/L), NaNO_3_ (3 g/L), KH_2_PO_4_ (0.5 g/L), KCl (0.5 g/L) in 50% sea water at pH 7.5. The media was prepared in 85 Erlenmeyer flasks (1 L) and incubated at 30 ± 2 °C for 14 days. The Czapek’s peptone liquid medium (12 L) containing biomalt (20 g/L) and peptone (5 g/L) in 50% sea water at pH 7.5 using 60 Erlenmayer flasks (1 L) and incubated at 30 ± 2 °C for two weeks.

Each old fermentation broth was separated from its fungal mat. The medium and fungal mycelia were diluted with distilled water to enable them to be easily blended using the sonication bath for further extraction by ethyl acetate (EtOAc). The resultant extracts from each culture medium were combined and dried using a rotavapour 40 °C water bath. The resultant crude extracts were chromatographed over silica gel column using *n*-hexane as starting non-polar eluent and gradually increasing the polarity using EtOAc as polar solvent in mixture eluent (10%, 20%, until 100% EtOAc), followed by 10%, 20% and 50% MeOH/EtOAc with respect to each crude extract TLC-reference during the fractionation. The combined fractions were further purified using preparative silica gel followed by Sephadex LH-20 with MeOH, MeOH/CHCl_2_, and MeOH/CHCl_2_/*n*-Hexane (2:3 and 1:1:1, *v*:*v*) and MeOH/CH_2_Cl_2_/*n*-hexane (1:1:1, *v*:*v*:*v*) as well as semi-preparative HPLC using a C18 column, eluted with acetonitrile/water (85:15, isocratic method, 1.2 mL/min) to give 21 pure compounds. Compounds **1**–**12** were identified from the biomalt-peptone culture extract and compounds **7** and **13**–**20** were identified from the Czapek’s peptone culture extract. 

### 3.6. Assay for Determination of HCV Protease Inhibitory Activity 

Samples of 2 μL of culture broth fungus extracts and isolated compounds dissolved in dimethyl sulfoxide (DMSO) were placed in each well of a 384-well microplate, then 8 μL of recHCV PR (0.5 μg/mL) were added, and the plate was briefly agitated. Finally, 10 μL of the freshly prepared substrate [Ac-Asp-Glu-Dap (QXLTM520)-Glu-Glu-Abu-COO-Ala-Ser-Cys(5-FAMsp)-NH_2_] were added with sequential rotational shaking. The reaction mixture was incubated at 37 °C for 30 min. The fluorimetric analyses were performed on an automated TECAN GENios plate reader (Männedorf, Switzerland) with excitation and emission wavelengths at 485 and 520 nm, respectively. Each sample was tested in triplicate. HCV PR inhibition (%) was calculated using the following equation:

% Inhibition = (*F*_substrate_ − *F*_testsample_) × 100/*F*_substrate_

where *F*_substrate_ is the fluorescence of the enzyme and substrate only, and *F*_testsample_ is the fluorescence of the assay mixture with the added sample.

### 3.7. Green Protease Assay

The active HCV PR sample of extracts and isolated compounds were dissolved in dimethyl sulfoxide (2.5 μL; final content, 10% *w*/*v*) and placed in the wells of a 384-well microplate. Then 17.5 μL of assay buffer and 2.5 μL of trypsin (0.1 U/μL) were added and the plate was briefly agitated. Finally, 2.5 μL of the freshly diluted protease substrate HiLyte Fuor TM 488-labeled casein were added under sequential rotary shaking and the mixture incubated at 37 °C for 30 min. The positive control was the soybean trypsin-chymotrypsin inhibitor. Inhibition was calculated as above for HCV PR assay.

## 4. Conclusions

From the current study, it is evident that the mycelia growth and subsequent production of bioactive metabolites by the endophytic fungus, *Fusarium equiseti*, isolated from the brown alga *Padina pavonica*, is influenced by various nutrient supplements in the culture media where the maximum production of HCV PR inhibitor metabolites was achieved in Czapek’s-peptone medium, with IC_50_ values ranging from 10 to 37 μM/mL. The combination of in vitro assay against HCV PR together with docking analysis allowed us to identify ω-hydroxyemodin (**14**) as anti-HCV PR. These results will probably be a promising scaffold for the further development of HCV protease inhibitors.

## Figures and Tables

**Table 1:** Antimicrobial potential of the culture extracts and isolated compounds from *F. equiseti*.

Sample	Gram-Positive Bacteria			Fungi
	*Staphylococcus aureus*	*Bacillus megaterium*	*Bacillus subtilis*	*Candida albicans*
Ethyl acetate extract (bi-malt culture)	10 *	11	18	13
Cyclo-l-Ala-l-Leu (**1**)	-	12	-	13
Cyclo(l-Pro-l-Val) (**2**)	-	11	18	9
Cyclo-(Phenylalanyl-Pro-Leu-Pro) (**5**)	9	10	-	12
17-Demethyl-2,11-dideoxy-rhizoxin (**6**)	12	8	-	19
3-*O*-β-Glucosylsitosterol (**9**)	9	14	11	10
Griseoxanthone C (**12**)	-	10	13	-
Ethyl acetate extract (Czapek culture)	15	17	9	19
Chrysophanol (**13**)	15	10	-	11
ω-Hydroxyemodin (**14**)	12	17	9	18
Cyclo(l-Tyr-l-Pro) (**15**)	11	8	-	13
Perlolyrine (**16**)	14	9	8	10
Cordycepin (**17**)	16	11	9	14
Ara-A (**18**)	10	12	12	-
Oxytetracycline (30 μg)	17	20	18	-

* Inhibition zone in mm.

**Table 2:** Inhibition of Hepatitis C Virus (HCV) NS3-NS4A protease by crude extracts and isolated compounds (**1**–**20**) of *F. equiseti*.

Biomalt-Peptone Culture Medium			Czapek’s (Cz)-Peptone Culture Medium		
Sample			IC_50_ [μM]		
	HCV Protease Inhibitory Activity	Trypsin Inhibitory Activity	Sample	HCV Protease Inhibitory Activity	Trypsin Inhibitory Activity
EtOAc extract (Biomalt)	56.0 ± 18 μg/mL	>100	EtOAc extract (Cz)	27.65 ± 2.2 μg/mL	88.89 ± 3.39 μg/mL
Cyclo-l-Ala-l-Leu (**1**)	58.33 ± 3.51	Nt	Chrysophanol (**13**)	>1000	Nt
Cyclo(l-Pro-l-Val) (**2**)	23.29 ± 1.23	54.21 ± 1.88	ω-Hydroxyemodin (**14**)	10.71 ± 2.3	48.52 ± 1.9
Thymine (**4**)	51.82 ± 2.49	Nt	Cyclo(l-Tyr-l-Pro) (**15**)	18.20 ± 1.7	37.91 ± 3.8
Cyclo-(Phenylalanyl-Pro-Leu-Pro) (**5**)	29.45 ± 1.98	226.21 ± 4.26	Perlolyrine (**16**)	37.89 ± 2.11	>1000
17-Demethyl-2,11-dideoxy-rhizoxin (**6**)	34.42 ± 1.44	144.67 ± 3.66	Cordycepin (**17**)	22.35 ± 3.12	>1000
Ergostra-5,7-dien-3β-ol (**8**)	77.14 ± 4.55	Nt	Ara-A (**18**)	24.53 ± 2.3	137.58 ± 2.49
3-*O*-β-Glucosylsitosterol (**9**)	76.56 ± 3.78	Nt	Communiol D (**19**)	>1000	Nt
5-Chloro-3,6-dihydroxy-2-methyl-1,4-benzoquinone (**11**)	35.15 ± 3.92	294.82 ± 2.87	Ethyl-*O*-β-glucoside (**20**)	>1000	Nt
Griseoxanthone C (**12**)	19.88 ± 1.35	51.33 ± 2.34			
HCV-I_2_	1.64 ± 0.5		HCV-I_2_	1.64 ± 0.5	
T-I		0.5	T-I		0.5

Note: **HCV-I_2_**, HCV NS3-NS4A protease inhibitor 2 (positive control for HCV PR); **T-I**, soybean trypsin-chymotrypsin inhibitor (positive control for trypsin); Nt, not tested. Compounds **3**, **7** and **10** were not tested.
